# Studying on the efficiency of higher education resource allocation and its influencing factors in the western China using DEA-Malmquist and Tobit models

**DOI:** 10.1371/journal.pone.0334090

**Published:** 2025-10-16

**Authors:** Ziyu Ye, Ribesh Khanal, Zhiqiang Cao

**Affiliations:** 1 School of Education Science, Guangxi Normal University for Nationalities, Chongzuo, Guangxi, China; 2 College of Economics and Management, China Three Gorges University, Yichang, Hubei, China; 3 School of Economics and management, Guangxi Normal University for Nationalities, Chongzuo, Guangxi, China; University of Johannesburg, SOUTH AFRICA

## Abstract

Optimizing higher education resource allocation in western China is vital for advancing national development through education and talent. This research covers the DEA Malmquist to examine the effectiveness of higher education materials distributed statically and vigorously within twelve provinces in the western part of China. It also studies the internal inequalities in resource distribution effectiveness and employs the Tobit model to identify which the main factors affecting the efficiency of higher education resource allocation. The primarily data sources from the China Education Yearbook (2011–2021). The findings indicate that the comprehensive technical efficiency (TE), pure technical efficiency (PTE), and scale efficiency (SE) have not reached the efficiency frontier in higher education resource allocation in western China. Conversely, the dynamic analysis reveals a decline in overall efficiency in resource allocation for higher education in the western region, with significant variations in efficiency levels among the provinces. Factors such as education expenditure, GDP per capita, total GDP, and the breadth of education significantly impact the efficiency of resource allocation for higher education in the western region. To improve this efficiency, it is essential to boost financial input into education, adjust resource allocation strategies, focus on matching educational quality with market demands, and implement dynamic monitoring and evaluation.

## 1. Introduction

China has explicitly committed to “providing education that satisfies the people.” This commitment prioritizes a people-centered approach, promoting high-quality development, equity, and cross-regional resource optimization. The belief that “education, science, technology, and talent serve as foundational and strategic pillars for the comprehensive construction of a socialist modernized nation” is emphasized. Accelerating a high-quality education system in western China is vital for sustainable regional development and advancing the nation’s talent, technological, and educational capabilities. Achieving this requires prioritizing equitable internal resource distribution in western China alongside inter-regional optimization, strengthening equity-focused policies, innovating development mechanisms, and advancing digital transformation.

**Fig 1:**
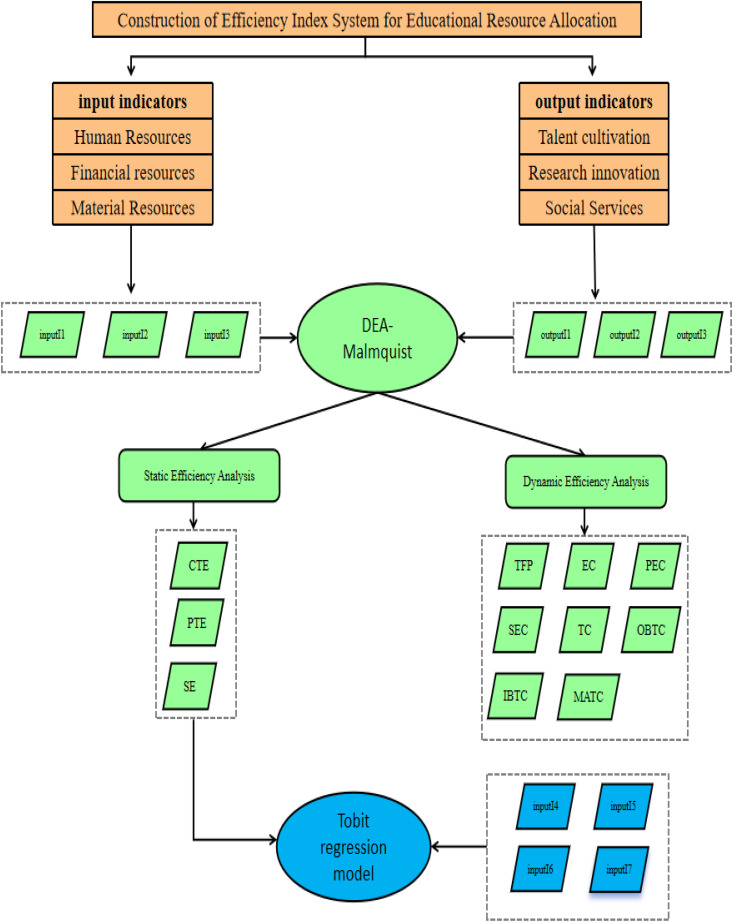
Methodological framework.

In this study, to reflect the overall efficiency of higher education resource allocation in the western region of China, we focus on the provincial level. This paper first constructs an index system for higher education resources to measure the input of human resources, financial resources, and material resources, as well as outputs including talent cultivation, scientific and technological innovation, and social services. The DEA-Malmquist model is used to determine both the static and dynamic efficiency of higher education resource allocation in each western province. In addition, this paper compares the dynamic changes in higher education resource allocation in the western region, identifies the main reasons for the low efficiency and potential improvement paths, and conducts a comparative analysis of regional efficiency both within China and globally. Finally, the truncated Tobit model is used to explore the variables affecting the efficiency of higher education resource allocation in the western region. Fig 1 shows the structure and flow chart of the article.

The remaining structure of this paper is as follows: Section 2 reviews the research field and methods related to higher education efficiency; Section 3 describes the model construction, indicator selection, and data sources; Section 4 presents an empirical analysis of higher education resource allocation efficiency in the western region; Section 5 examines the factors influencing efficiency; Section 6 presents the limitations and future research directions.

## 2. Literature review

The education system is a complex, multi-layered structure characterized by frequent inputs and outputs. Inputs in higher education include manpower, materials, and financial resources, while outputs comprise talent cultivation, research innovation, and social services. However, the causal relationship between inputs and outputs remains unclear, necessitating evaluation of not only suitability and fairness but also of the significance and efficacy of resource distribution in western China. At present, evaluating the effectiveness of higher education resources has emerged as a key focus of academic inquiry. The main methodological approach is Data envelopment Analysis (DEA), which offers distinct advantages for examining multiple inputs and outputs without requiring predefined production. These investigations have provided both theoretical and empirical support for enhancing resource allocation efficiency, fostering educational equity, and improving the quality of education.

Many scholars have conducted research on evaluating the efficiency evaluation of higher education research allocation. Zhang Qiang (2022) conducted both static and dynamic efficiency evaluations of resource allocation in higher education across the Beijing-Tianjin-Hebei and Yangtze River Delta regions, using DEA-BCC and Malmquist index models. The study revealed regional disparities and highlighted changes in efficiency trends over time [1]. You Li (2021) applied the super-efficiency DEA-Malmquist approach to analyze the dynamic efficiency of higher education resource distribution across 31 provinces in China. The findings indicated that shifts in technical efficiency were the main drivers of overall allocation efficiency, which generally showed an upward trend [2]. Jiang Yucheng (2020) evaluated the efficiency of China’s resource allocation in higher education using the DEA-CCR model and Malmquist index, identifying a significant gap caused by lower efficiency in eastern provinces compared to central and western regions, along with clear waste in educational inputs [3]. Ye Qianlin (2018) and Li Yuanjing (2014) evaluated and conducted spatial correlation analyses on the efficiency of higher education resource allocation across China’s 31 provinces using DEA methods and spatial data analysis, respectively. Further analysis using the Tobit model provided insights into the main factors affecting efficiency, offering a basis for policymaking [4,5]. Zhou Xiaogang (2022) and Zhang Hong (2015) evaluated China’s educational efficiency using three-stage DEA and super-efficiency DEA, respectively, finding high overall efficiency with significant regional disparities [6,7]. Guo, H (2023), and Yuanzhi Guo (2024) focused on the issue of regional inequality in educational development in India and China, respectively, revealing key factors for promoting educational fairness through DEA and Tobit model analyses [8,9], with Yuanzhi Guo’s research highlighting the impact of large-scale population migration and rapid industrialization on educational resource allocation [9]. Bo Yang (2024) constructed a comprehensive evaluation model based on the traits of the ethnic regions in China to explore ways to increase compulsory education investment [10].

Moreover, researchers including Samira El Gibari (2022), Ma Junfeng (2025), Li Ning et al. (2024), and Dong Junyan et al. (2024) have employed techniques such as the super-efficiency DEA Malmquist model, the DEA-BCC model, and the three-stage DEA, often in conjunction with Tobit regression, to evaluate the efficiency of educational resource allocation and examine its influencing factors [11–14]. Additionally, scholars like Yao Hao (2023), Liu Baotao (2025), Agasisti (2021), Zhao Qingnian (2024), and Liu (2024) have conducted comprehensive studies on the micro-level efficiency of resource allocation in graduate education, higher education innovation, a system-level resource deployment, and transportation system. These studies have further advanced the application of DEA models and their extended variants [15–19].

Traditional DEA models primarily focus on static efficiency and often overlook the dynamic aspects of higher education resource allocation. Moreover, existing research has given limited attention to western China, with most prior studies concentrating on nationwide evaluations. To fill this gap, this study applies the DEA-Malmquist approach to assess both the static and dynamic efficiency of higher education resource allocation in western China. In addition, it employs the Tobit regression model to analyze the key factors that impact this efficiency. The findings of this study strengthen the theoretical framework surrounding resource allocation in higher education and offer practical recommendations for improving allocation strategies, thereby supporting the balanced and sustainable development of higher education in the western region.

## 3. Model construction, indicator selection and data sources

### 3.1. DEA-Malmquist

DEA is a nonparametric method designed to evaluate the relative efficiency of comparable decision-making units by analyzing multiple input and output indicators. Originally introduced in 1978 by Charnes, Cooper, and Rhodes, the initial DEA model, later referred to as the CCR model, laid the foundation for measuring technical efficiency through relative comparisons [8]. Due to its flexibility and straightforward structure, DEA has become especially useful in evaluating performance in contexts involving multiple inputs and outputs, offering distinct advantages in such analyses. As a result, its application scope has rapidly expanded, involving numerous fields such as education, agriculture, environment, macroeconomics, finance, taxation, healthcare, sports, public transportation, power, postal services, telecommunications, logistics, military, and business management.

According to the different assumptions about returns to scale, DEA models are commonly divided into two types: the CCR model and the BCC model. The CCR (Charnes, Cooper, and Rhodes) model assumes constant returns to scale (CRS), and the resulting technical efficiency includes both pure technical efficiency and scale efficiency, which is why it is often referred to as comprehensive technical efficiency. On the other hand, the BCC (Banker, Charnes, and Cooper) model is based on variable returns to scale (VRS), and the technical efficiency obtained excludes the effect of scale, representing what is known as pure technical efficiency (PTE). This study assumes that the higher education system in western China operates under variable returns to scale. Based on this, the BCC model is applied to explore how to minimize input levels given a set level of output, with the aim of identifying the minimum value of θ. The specific form of the BCC model is shown below.


$$\begin{array}{c} \min\theta \\ s.t\;\sum_{j=1}^{n}X_{j}\lambda_{j}\leq \theta X_{0},\\ \;\;\sum_{j=1}^{n}Y_{j}\lambda_{j}\geq Y_{0},\\ \;\;\sum_{j=1}^{n}\lambda_{j}=1\\ \;\;\lambda_{j}\geq 0,\;j=1,\cdots ,n \end{array}$$ (1)

In the above equation, θ represents the overall efficiency score of the decision-making unit (DMU). When θ is less than 1, the DMU is considered inefficient under DEA analysis. When θ equals 1, the DMU is deemed DEA efficient. The variables X_j_ and Y_j_ denote the input and output values for the type j, respectively. The symbol λ indicates the weight assigned to the peer DMUs in constructing a reference point, and k is the total number of DMUs being evaluated.

The BCC model was introduced to assess the technical efficiency of DMUs within the framework of Variable Returns to Scale (VRS) production technology. In addition to measuring technical efficiency, the BCC model also enables the evaluation of scale efficiency. When production operates under VRS, the efficiency score obtained from the Constant Returns to Scale (CRS) model reflects not only pure technical efficiency but also includes scale effects. By comparing the CRS and VRS efficiency scores, one can isolate the value of scale efficiency (SE). In this context, the VRS-based efficiency value, also referred to as pure technical efficiency (PTE), represents the DMU’s ability to convert inputs into outputs without the influence of scale.


$$Scale\;Efficiency(SE)=\frac{Compresive\;Techniacl\;Efficiency(TE)}{Pure\;Technical\;Efficiency(PTE)}$$ (2)

When working with panel data containing observations across multiple time periods, a widely used method for assessing productivity changes and distinguishing between the effects of technical efficiency and technological advancement is the Malmquist Total Factor Productivity (TFP) index. The theoretical foundation of this index originates from the work of Malmquist (1953), and productivity indices based on this approach are commonly known as Malmquist indices. This index measures productivity variation between two time periods by comparing the relative performance of the later period to the earlier one.

Färe et al. (1992) were the first to apply DEA in the calculation of the Malmquist index. Their framework decomposes the index into two key components: the first is the change in technical efficiency (EC), which captures how well a DMU improves its use of resources over time; the second is technological change (TC), which represents shifts in the production frontier, reflecting innovation or technological improvement.


$$\begin{array}{c} M(x^{t+1},y^{t+1},x^{t},y^{t})={\left(\frac{E^{t}(x^{t+1},y^{t+1})}{E^{t}(x^{t},y^{t})}*\frac{E^{t+1}(x^{t+1},y^{t+1})}{E^{t+1}(x^{t},y^{t})}\right)}^{\frac{\text{1}}{\text{2}}}\\ =\frac{E^{t+1}(x^{t+1},y^{t+1})}{E^{t}(x^{t},y^{t})}*{\left(\frac{E^{t}(x^{t},y^{t})}{E^{t+1}(x^{t},y^{t})}\frac{E^{t}(x^{t+1},y^{t+1})}{E^{t+1}(x^{t+1},y^{t+!})}\right)}^{\frac{\text{1}}{\text{2}}}=EC*TC \end{array}$$ (3)

In the above equation, $E^{t}(x^{t},y^{t})$ and $E^{t+\text{1}}(x^{t+\text{1}},y^{t+\text{1}})$ denote the technical efficiency values of k in two periods, respectively. Building on the decomposition proposed by Färe et al. (1992), their later work in 1994 further divided the technical efficiency change (EC) into two distinct components: pure efficiency change (PEC), also referred to as pure technical efficiency change, and scale efficiency change (SEC). These two elements reflect the influence of internal management practices and scale economies on the production frontier. A value greater than one for either component suggests a positive contribution to the expansion of the production frontier, whereas a value less than one indicates a negative effect on productivity growth [10].


MI=EC*TC=PEC*SEC*TC (4)

$$\begin{array}{c} PEC=\frac{E{\mathrm{\backslash nolimits}}^{t+1}(x{\mathrm{\backslash nolimits}}^{t+1},y{\mathrm{\backslash nolimits}}^{t+1}|VRS)}{E{\mathrm{\backslash nolimits}}^{t}(x{\mathrm{\backslash nolimits}}^{t},y{\mathrm{\backslash nolimits}}^{t}|VRS)}\\ SEC=\frac{E{\mathrm{\backslash nolimits}}^{t+1}(x{\mathrm{\backslash nolimits}}^{t+1},y{\mathrm{\backslash nolimits}}^{t+1}|CRS)}{E{\mathrm{\backslash nolimits}}^{t+1}(x{\mathrm{\backslash nolimits}}^{t+1},y{\mathrm{\backslash nolimits}}^{t+1}|VRS)}\times \frac{E{\mathrm{\backslash nolimits}}^{t}(x{\mathrm{\backslash nolimits}}^{t},y{\mathrm{\backslash nolimits}}^{t}|VRS)}{E{\mathrm{\backslash nolimits}}^{t}(x{\mathrm{\backslash nolimits}}^{t},y{\mathrm{\backslash nolimits}}^{t}|CRS)} \end{array}$$ (5)

VRS refers to variable returns to scale, while CRS stands for constant returns to scale. Technological change (TC) can be further broken down into three components: Output Biased Technological Change (OBTC), Input Biased Technological Change (IBTC), and the Magnitude of Technological Change (MATC). Specifically, OBTC (t, t + 1) reflects output-oriented bias in technological progress, indicating deviations caused by changes in output between time periods t and t + 1. Similarly, IBTC (t, t + 1) captures input-oriented bias, representing variations due to changes in input over the same periods. MATC (t, t + 1) denotes the overall extent of technological change, with values above 1 signifying progress through a forward shift in the production frontier, and values below 1 indicating technological decline, or a backward movement of the frontier.


TC=OBTC*IBTC*MATC (6)

### 3.2. Tobit model

This paper employs the Tobit method to conduct a regression analysis on the influencing factors of resource allocation efficiency values, with the efficiency value as the dependent variable. The Tobit model is applicable to data where the dependent variable is truncated or censored during data processing and is related to the independent variables. It is also known as the sample selection model or the limited dependent variable model and is a type of regression model where the dependent variable is restricted. The concept was first proposed by Nobel laureate in economics, James Tobit, and has since been continuously developed and improved by numerous economists [19,20]. Since the resource allocation efficiency values calculated using the DEA model fall within the range of [0, 1], they are truncated data and related to the explanatory variables. The estimation results obtained using the ordinary least squares method are often biased and inconsistent. By using the Tobit model based on the principle of maximum likelihood estimation for the analysis of influencing factors, the deviations caused by data restrictions and large data variability can be minimized. Therefore, this paper adopts the Tobit model for parameter estimation. For the i-th region, the specific estimation form of the Tobit model is as follows:


$$\begin{array}{c} y_{i}=\beta_{\text{0}}+\sum_{j=1}^{k}\beta_{j}x_{ij}+\varepsilon_{i}\\ y_{i}=y_{i}^{*},ify_{i}^{*}>0\\ y_{i}=0,ify_{i}^{*}\leq 0\\ \varepsilon_{i}\sim N(0(\delta^{\text{2}}) \end{array}$$ (7)

Among them, the explained variable *y*_*i*_ is the value of the higher education allocation efficiency of each province in the western region in the Tobit model, which is *β* the parameter to be estimated. $y_{i}^{*}$ is the actual value of the higher education allocation efficiency of each province in the western region calculated based on the DEA model, and *x*_*ij*_ is an influencing factor of the resource allocation efficiency of each province in the western region.

### 3.3. Selection of indicators

The evaluation of resource allocation efficiency in higher education must be fundamentally grounded in three core dimensions: regional development disparities, the multifunctionality of educational functions, and resource scarcity. Within this theoretical framework, the present study integrates growth pole theory (Perroux, 1950), human capital theory (Schultz, 1961), and the tripartite mission theory of higher education (Clark, 1983) as theoretical cornerstones for constructing the indicator system. This integrated approach establishes an evaluative framework that reconciles theoretical rigor with contextual adaptability to the western region. In strict adherence to the normative requirements of the Data Envelopment Analysis (DEA) model—specifically, that the number of Decision-Making Units (DMUs) must satisfy the condition n ≥ m × s (where m and s denote the quantities of input and output indicators, respectively)—we have designed the following indicator system, tailored to the empirical realities of the 12 western provinces., as shown in Table 1.

**Table 1:** Evaluation index of efficiency of higher education resource allocation in western China.

Input	Human Resources	The number of full-time teachers in higher education is X1
	Financial resources	Expenditure on education X2
	Material Resources	The total value of fixed assets of colleges and universities is X3
Output	Talent cultivation	This is equivalent to Y1 in the number of students enrolled Y2
	Research innovation	Colleges and universities publish academic papers Y2
	Social Services	R&D Achievement Application and Technology Service Project Y3

#### 3.3.1. Input indicators.

Input indicators focus on resource scarcity and spatial agglomeration, selecting human resources, financial resources, and material resources as key metrics.

Human capital theory (Schultz, 1961) posits that teachers constitute the core capital of knowledge production, with their scale directly impacting the return on educational investment [21]. Therefore, the human resources indicator is selected, measured by the number of full-time higher education faculty. As full-time faculty form the foundation of talent cultivation and most western universities face faculty shortages with high student-teacher ratios, using full-time faculty numbers as a human resources metric aligns with the actual conditions of western institutions.

Growth pole theory (Perroux, 1950) suggests that resources concentrate in core cities, leaving peripheral regions underinvested, with central transfer payments exceeding 60% supporting western education [22]. Consequently, financial resources are chosen as an indicator, representing crucial higher education funding and serving as the economic foundation for sustainable development in western higher education. Based on existing research, educational expenditure is adopted as the metric for financial resources.

The polarization effect in growth pole theory reveals a significant resource gap between core and peripheral areas, with provincial capital universities possessing equipment values several times higher than prefecture-level cities. Thus, material resources are selected as an encompassing term for various physical assets in education. This study employs the fixed asset value of higher education institutions as the measurement indicator for material resources.

#### 3.3.2. Output indicators.

The output indicators correspond to the multifaceted functions of education and regional needs, selecting talent cultivation, research innovation, and social service. The Triangle of Coordination Theory (Burton R. Clark, 1983) posits that higher education institutions must synergistically fulfill three core functions [23]: talent cultivation, research innovation, and social service. This theory directly informs the construction of the output indicator system within the resource allocation efficiency evaluation framework.

Talent cultivation, cultivating high-quality talents is the goal of talent cultivation in institutions of higher education, and there are mainly undergraduate, master’s degree, and doctoral degrees at the cultivation level of higher education. Due to the different cultivation times and costs, this paper adopts the number of students in the folding (number of students in the undergraduate * 1 + master’s degree students * 1.5 + doctoral degree students * 2) as the output indicator of the dimension of talent cultivation.

Research innovation in higher education encompasses both basic research, where universities serve as China’s primary hubs for scientific advancement and foundational research support, and applied research, which focuses on societal contributions. Hence, this paper chooses academic papers and scientific works as key metrics to measure the output of fundamental research, considering them the most important output of basic research.

Social services, R&D results application and scientific and technological service programs are the direct performance of universities to serve the community. Therefore, this paper adopts R&D results application and scientific and technological service projects as the indicators of social service.

### 3.4. Data sources and processing

The research sample spans the period from 2011 to 2021 and encompasses 12 provinces (including autonomous regions) in western China. The primary data sources utilized in this study include the *China Education Yearbook (2011–2021), the China City Statistical Yearbook (2012–2022), the Compilation of Statistics on Higher Education Science and Technology (2012–2022)*, and *the China Education Finance Yearbook (2012–2022)*. Where missing data points had appeared, interpolation techniques were used to ensure the completeness and consistency of the dataset, enabling a comprehensive and rigorous analysis.

## 4. Empirical analysis of resource allocation efficiency of higher education in western China

### 4.1. Static efficiency analysis

Using panel data from 12 provinces in western China covering the period 2011–2021, this study applies MAXDEA software to evaluate the efficiency of higher education resource allocation in the region. As presented in Table 2, the results show that the average values for comprehensive technical efficiency (TE), pure technical efficiency (PTE), and scale efficiency (SE) range between 0.9100 and 1.000, reflecting a generally high level of efficiency. Nonetheless, it is important to note that neither of the provinces have fully reached the efficiency of the frontier, suggesting that there is still considerable room for further enhancement and optimization in the distribution of higher education resources, as shown in Table 2.

**Table 2:** Measurement of the efficiency of higher education resource allocation in western China.

Regions	Efficient Type	2011	2012	2013	2014	2015	2016	2017	2018	2019	2020	2021
Gansu	CTE	1.000	1.000	1.000	1.000	1.000	1.000	1.000	1.000	0.946	0.907	1.000
	PTE	1.000	1.000	1.000	1.000	1.000	1.000	1.000	1.000	0.983	0.968	1.000
	SE	1.000	1.000	1.000	1.000	1.000	1.000	1.000	1.000	0.962	0.937	1.000
	RS	—	—	—	—	—	—	—	—	irs	irs	—
Guangxi	CTE	1.000	1.000	1.000	1.000	1.000	1.000	1.000	1.000	1.000	1.000	1.000
	PTE	1.000	1.000	1.000	1.000	1.000	1.000	1.000	1.000	1.000	1.000	1.000
	SE	1.000	1.000	1.000	1.000	1.000	1.000	1.000	1.000	1.000	1.000	1.000
	RS	—	—	—	—	—	—	—	—	—	—	—
Guizhou	CTE	1.000	1.000	1.000	0.969	0.894	0.925	0.996	0.996	1.000	0.922	0.968
	PTE	1.000	1.000	1.000	0.972	0.895	0.925	1.000	1.000	1.000	0.946	1.000
	SE	1.000	1.000	1.000	0.998	0.999	0.999	0.996	0.996	1.000	0.974	0.968
	RS	—	—	—	irs	irs	irs	drs	drs	—	drs	drs
Inner Mongolia	CTE	0.797	0.801	0.787	0.814	0.675	0.700	0.888	0.888	0.829	0.706	0.829
	PTE	0.800	0.804	0.789	0.821	0.698	0.746	0.890	0.890	0.867	0.778	0.857
	SE	0.996	0.997	0.998	0.991	0.967	0.938	0.998	0.998	0.956	0.907	0.968
	RS	drs	drs	irs	irs	irs	irs	irs	irs	irs	irs	irs
Lingxia	CTE	1.000	1.000	1.000	1.000	1.000	0.744	0.752	0.752	0.700	0.771	0.760
	PTE	1.000	1.000	1.000	1.000	1.000	1.000	1.000	1.000	1.000	1.000	1.000
	SE	1.000	1.000	1.000	1.000	1.000	0.744	0.752	0.752	0.700	0.771	0.760
	RS	—	—	—	—	—	irs	irs	irs	irs	irs	irs
Qinghai	CTE	1.000	1.000	1.000	1.000	1.000	1.000	1.000	1.000	1.000	1.000	1.000
	PTE	1.000	1.000	1.000	1.000	1.000	1.000	1.000	1.000	1.000	1.000	1.000
	SE	1.000	1.000	1.000	1.000	1.000	1.000	1.000	1.000	1.000	1.000	1.000
	RS	—	—	—	—	—	—	—	—	—	—	—
Shaanxi	CTE	1.000	1.000	1.000	1.000	1.000	1.000	1.000	1.000	1.000	1.000	1.000
	PTE	1.000	1.000	1.000	1.000	1.000	1.000	1.000	1.000	1.000	1.000	1.000
	SE	1.000	1.000	1.000	1.000	1.000	1.000	1.000	1.000	1.000	1.000	1.000
	RS	—	—	—	—	—	—	—	—	—	—	—
Sichuan	CTE	1.000	0.985	0.961	0.942	0.983	1.000	1.000	1.000	1.000	1.000	1.000
	PTE	1.000	1.000	1.000	1.000	1.000	1.000	1.000	1.000	1.000	1.000	1.000
	SE	1.000	0.985	0.961	0.942	0.983	1.000	1.000	1.000	1.000	1.000	1.000
	RS	—	drs	drs	drs	drs	—	—	—	—	—	—
Tibet	CTE	0.994	0.854	0.841	0.808	0.689	0.693	0.702	0.702	0.682	0.620	0.570
	PTE	1.000	1.000	1.000	1.000	1.000	1.000	1.000	1.000	1.000	1.000	1.000
	SE	0.994	0.854	0.841	0.808	0.689	0.693	0.702	0.702	0.682	0.620	0.570
	RS	irs	irs	irs	irs	irs	irs	irs	irs	irs	irs	irs
Xinjiang	CTE	1.000	1.000	1.000	1.000	1.000	1.000	1.000	1.000	1.000	1.000	1.000
	PTE	1.000	1.000	1.000	1.000	1.000	1.000	1.000	1.000	1.000	1.000	1.000
	SE	1.000	1.000	1.000	1.000	1.000	1.000	1.000	1.000	1.000	1.000	1.000
	RS	—	—	—	—	—	—	—	—	—	—	—
Yunnan	CTE	0.909	0.887	0.858	0.840	0.853	0.894	0.882	0.882	0.924	0.913	0.972
	PTE	0.976	0.915	0.893	0.870	0.906	0.947	0.950	0.950	0.931	0.919	1.000
	SE	0.931	0.970	0.961	0.966	0.942	0.945	0.928	0.928	0.993	0.994	0.972
	RS	drs	drs	drs	drs	drs	drs	drs	drs	drs	drs	drs
Chongqing	CTE	0.938	0.974	0.940	0.956	0.929	0.964	0.964	0.964	0.938	0.938	0.987
	PTE	0.939	0.976	0.943	0.961	0.932	0.965	0.966	0.966	0.967	0.954	0.988
	SE	0.999	0.998	0.997	0.994	0.997	0.999	0.998	0.998	0.970	0.984	0.999
	RS	drs	irs	irs	irs	irs	drs	drs	drs	irs	irs	irs
Averages	CTE	0.970	0.958	0.949	0.944	0.919	0.910	0.932	0.932	0.918	0.898	0.924
	PTE	0.976	0.975	0.969	0.969	0.953	0.965	0.984	0.984	0.979	0.964	0.987
	SE	0.993	0.984	0.980	0.975	0.965	0.943	0.948	0.948	0.939	0.932	0.936

Note: Comprehensive Technical Effectiveness (CTE), Pure Technical Effectiveness (PTE), Scale Effectiveness (SE), RS represent Remuneration for scale, drs, and irs represent the constant return to scale, the increasing return to scale, and the decreasing return to scale, respectively.

#### 4.1.1. Comprehensive technical efficiency.

Over the period from 2011 to 2021, there were notable differences in the comprehensive technical efficiency values of higher education resource allocation across the western provinces of China. Provinces such as Guangxi, Qinghai, Shaanxi, and Xinjiang consistently recorded a comprehensive technical efficiency value of 1.000, indicating DEA effectiveness. In contrast, Gansu, Guizhou, Sichuan, Yunnan, and Chongqing demonstrated varying degrees of progress towards an efficiency value of 1.000 but did not achieve full DEA effectiveness. Meanwhile, Ningxia, Inner Mongolia, and Tibet have in recent years moved further away from achieving DEA effectiveness, showing a decline in their comprehensive technical efficiency values. This divergence highlights disparities in the efficiency of higher education resource allocation across the region and underscores the need for targeted efforts to enhance efficiency, particularly in provinces that are lagging behind.

#### 4.1.2. Pure technical efficiency analysis.

Guangxi, Qinghai, Shaanxi, and Xinjiang all maintain a pure technical scale efficiency of 1.000, indicating consistent effectiveness. Gansu and Sichuan occasionally show a scale technical efficiency below 1.000 but generally remain close to effective status. Conversely, Guizhou and Ningxia show the opposite trend, maintaining overall inefficiency. Tibet displays consistently ineffective pure technical efficiency with increasing returns to scale, while Yunnan shows inefficiency with decreasing returns to scale. Inner Mongolia and Chongqing also show ineffective scale technical efficiency, with a mix of increasing and declining returns to scale.

Guangxi, Ningxia, Qinghai, Shaanxi, Sichuan, Tibet, and Xinjiang have consistently demonstrated effective levels of pure technical efficiency throughout the evaluation period. In contrast, Gansu, Guizhou, Yunnan, and Chongqing have shown relatively stable performance, with pure technical efficiency values generally above 0.900, indicating partial DEA effectiveness in certain years. However, Inner Mongolia has exhibited lower levels of pure technical efficiency along with significant fluctuations, suggesting substantial room for improvement in this region.

#### 4.1.3. Analysis of scale efficiency.

Guangxi, Qinghai, Shaanxi, and Xinjiang’s maintain scale efficiency is 1.000 effective state reflecting an effective state. Gansu and Sichuan occasionally exhibit scale efficiency below 1.000 but overall remain close to effective. In contrast, Guizhou and Ningxia consistently remain ineffective overall. Tibet shows persistent inefficiency in scale efficiency with increasing returns to scale, while Yunnan displays inefficiency with decreasing returns to scale. In Inner Mongolia and Chongqing, scale efficiency is also non-effective, with analysis of returns to scale revealing both increasing and decreasing trends.

### 4.2. Dynamic efficiency analysis

**Fig 2:**
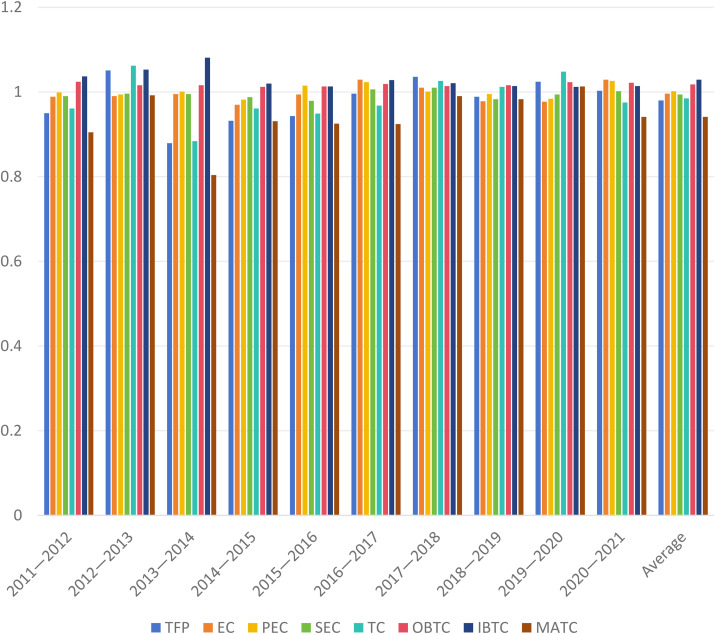
Decomposition of Malmquist index of higher education resource allocation in western China.

Building on the static efficiency analysis, this study employs the Malmquist index model to conduct a dynamic evaluation of higher education resource allocation efficiency in western China. The analysis yields several key indices, including the Total Factor Productivity index (TFP), Efficiency Change index (EC), Pure Efficiency Change index (PEC), Scale Efficiency Change index (SEC), Technological Change index (TC), Output Biased Technological Change (OBTC), Input Biased Technological Change (IBTC), and the Magnitude of Technological Change (MATC). The detailed results of these indices are presented in Table 3 and Fig 2.

**Table 3:** Decomposition of Malmquist index of higher education resource allocation in western China.

Years	TFP	EC	PEC	SEC	TC	OBTC	IBTC	MATC
2011—2012	0.950	0.989	0.999	0.990	0.961	1.024	1.037	0.905
2012—2013	1.051	0.990	0.994	0.996	1.062	1.016	1.053	0.992
2013—2014	0.879	0.995	1.001	0.995	0.884	1.016	1.081	0.804
2014—2015	0.932	0.970	0.982	0.988	0.961	1.012	1.020	0.931
2015—2016	0.943	0.994	1.015	0.979	0.949	1.013	1.013	0.925
2016—2017	0.996	1.029	1.023	1.006	0.968	1.019	1.028	0.924
2017—2018	1.036	1.010	1.001	1.010	1.026	1.014	1.021	0.990
2018—2019	0.989	0.978	0.995	0.983	1.012	1.016	1.014	0.983
2019—2020	1.024	0.977	0.984	0.994	1.048	1.023	1.012	1.013
2020—2021	1.003	1.029	1.026	1.002	0.975	1.022	1.014	0.941
Average	0.980	0.996	1.002	0.994	0.985	1.018	1.029	0.941

According to Table 3 and Fig 2, The impact of fiscal policy on the efficiency of higher education resource allocation in the western region shows a clear cyclical characteristic. From the efficiency evolution trajectory from 2010 to 2020, fluctuations in fiscal investment directly affect the trend of changes in total factor productivity. The decline in efficiency from 2012 to 2013 was due to the impact of economic conditions. As the global economic crisis resurfaced in 2012, the world economy began with a low opening and a high rise, which was widely believed at the beginning of the year and ended with a parabolic decline by year’s end. The global economic recovery in 2012 was sluggish, and China’s GDP growth rate was 7.7%, making it the slowest year for China’s economic growth since entering the 21st century. Funding for higher education in the western region mainly relied on fiscal appropriations, which led to significant resource constraints for western universities with a dependence rate of over 70%. So, from 2012 to 2013, the decrease in fiscal appropriations and the shortage of education funds led to a decrease in the efficiency of resource allocation in higher education.

The sustained improvement in efficiency after 2016 can be attributed to the implementation of special policies such as the “Central and Western Higher Education Revitalization Plan”, which explicitly required the central government to increase the average annual growth rate of student funding for western universities by no less than 8%, while strengthening local matching investment through the “provincial and ministerial joint construction” mechanism. This differentiated fiscal policy produced significant regional regulatory effects: on the one hand, institutional arrangements such as the “Support for Enrollment Cooperation Plan in the Central and Western Regions”, provided additional enrollment slots and supporting funds; on the other hand, compensatory policies such as the “Special Plan for Targeted Enrollment in Poverty stricken Areas” improved the quality of student intake in the western region. The “Action Plan for University Science and Technology Innovation to Serve Western Development” implemented by the Ministry of Education in 2018, increased the proportion of research funding tilted towards the western region to 15%. This institutionalized fiscal transfer mechanism effectively broke the “Matthew Effect”, resulting in a continuous increase in total productivity of western universities from 2016 to 2020, significantly faster than the national average level. These pieces of evidence indicate that China’s legislative and specialized fiscal transfer payment system can indeed implement precise regulation of the shortcomings in higher education development in different regions, thereby changing the evolution path of resource allocation efficiency.

**Fig 3:**
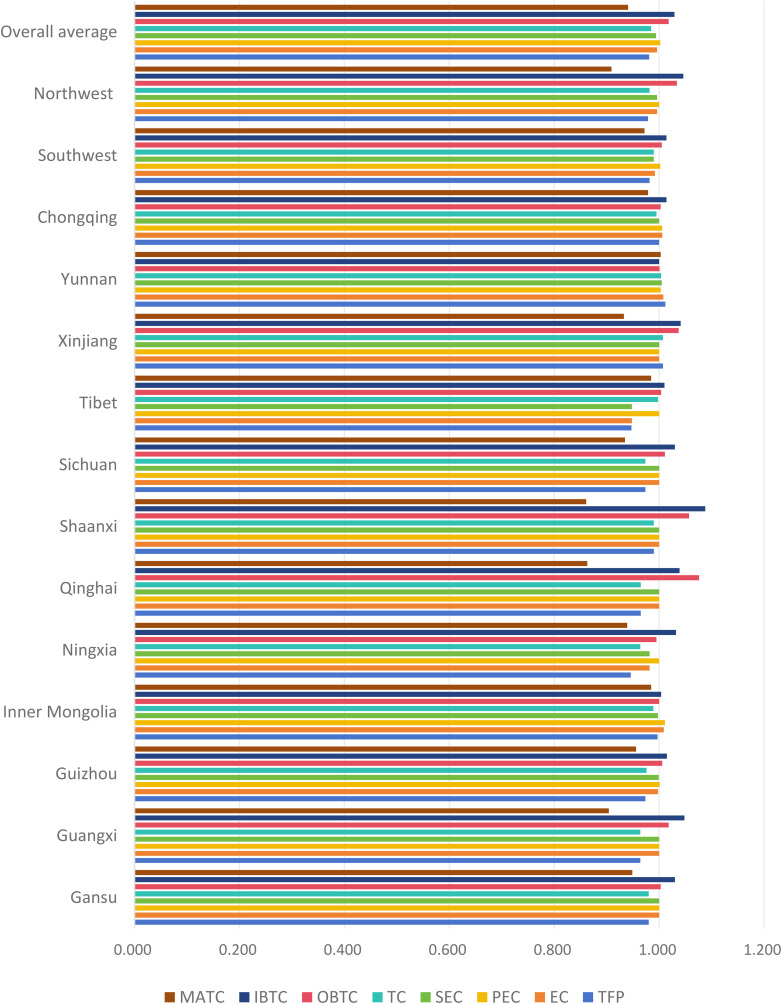
Changes in the average efficiency of higher education resource allocation in the western China.

Similarly, this study utilizes the Malmquist index to assess the efficiency of higher education resource allocation across various provinces in western China. As shown in Table 4 and Fig 3, the average Total Factor Productivity (TFP) value is 0.9812, which falls below the benchmark value of 1, indicating that overall resource allocation in the region does not achieve DEA efficiency standards. This points to relatively low efficiency levels in higher education resource utilization.

**Table 4:** Changes in the average efficiency of higher education resource allocation in the western China.

Province	TFP	EC	PEC	SEC	TC	OBTC	IBTC	MATC
Gansu	0.980	1.000	1.000	1.000	0.980	1.003	1.030	0.949
Guangxi	0.964	1.000	1.000	1.000	0.964	1.018	1.048	0.904
Guizhou	0.974	0.998	1.001	0.999	0.976	1.006	1.015	0.956
Inner Mongolia	0.997	1.009	1.011	0.998	0.989	1.000	1.004	0.985
Ningxia	0.946	0.982	1.000	0.982	0.964	0.995	1.032	0.939
Qinghai	0.965	1.000	1.000	1.000	0.965	1.076	1.039	0.863
Shaanxi	0.990	1.000	1.000	1.000	0.990	1.057	1.088	0.861
Sichuan	0.974	1.000	1.000	1.000	0.974	1.011	1.030	0.935
Tibet	0.947	0.948	1.000	0.948	0.998	1.004	1.010	0.985
Xinjiang	1.007	1.000	1.000	1.000	1.007	1.037	1.041	0.933
Yunnan	1.012	1.008	1.003	1.005	1.004	1.001	1.000	1.003
Chongqing	1.000	1.006	1.006	1.000	0.995	1.003	1.014	0.979
Southwest	0.982	0.992	1.002	0.990	0.990	1.005	1.014	0.972
Northwest	0.979	0.996	1.000	0.996	0.982	1.034	1.046	0.909
Overall average	0.981	0.996	1.002	0.994	0.985	1.018	1.029	0.941

Additionally, the results reveal a clear pattern of regional disparity, with approximately two-thirds of the provinces recording TFP values below 1. Notably, the total factor productivity (TFP = 0.9819) of the five southwestern provinces (Sichuan, Guizhou, Yunnan, Tibet, Chongqing) is higher than that of the five northwestern provinces (Shaanxi, Gansu, Qinghai, Ningxia, Xinjiang) (TFP = 0.9788). Furthermore, examining the index decomposition, on one hand, both the efficiency changes index (EC) and the technological change index (TC) hindered the overall efficiency improvement, contributing negatively to the overall efficiency. This suggests that in some areas of the western China, the input-output efficiency in higher education remains relatively underdeveloped. On the other hand, a more thorough analysis of the efficiency of technological advancement shows that input-biased technological change (IBTC) contributes more to technological advancement than output-biased technological change (OBTC). Additionally, the magnitude of technological change index (MATC) has a negative impact on technological progress, suggesting that technological advancement in western China’s higher education leans more toward input-focused improvements while lacking direction in output-oriented development and overall technological change magnitude.

Looking at the index decomposition, Xinjiang exhibits the highest total factor productivity (TFP = 1.0067), primarily due to technological advancements, while Ningxia displays the lowest total factor productivity (TFP = 0.9461) resulting from a decline in technological progress. In terms of efficiency change index, Guizhou, Ningxia, and Tibet show values less than 1, indicating some improvement in technical efficiency in most western provinces under the assumption of constant returns to scale. Further decomposition of the efficiency changes index reveals that in Guizhou, Ningxia, and Tibet, their index of efficiency change is less than 1.000 primarily due to a scale efficiency change index below 1, indicating uneconomical expenditure scales. Analyzing the pure technical efficiency change index, all twelve provinces in the western China show indices greater than or equal to 1.000, suggesting relatively sound higher education management systems in the western China. Looking at the technological progress index, Xinjiang and Yunnan exhibit indices greater than 1.000, indicating strong technological innovation capabilities in higher education for these provinces. However, the technological progress index for other provinces is less than 1.000, indicating a need to enhance technological innovation capabilities. Further breakdown of the technological progress index reveals that, except for Yunnan, the magnitude of technological change index (MATC) for all western provinces is less than 1.000, implying that technological progress in higher education in western China is primarily constrained by the magnitude of technological change. Although technological changes exist in the western China, the overall magnitude of change is relatively small, necessitating closer alignment with technological advancements of the times.

### 4.3. Regional comparative analysis

#### 4.3.1. Domestic regional efficiency comparison.

An analysis of higher education resource allocation efficiency across China’s four major regions (2011–2021) reveals distinct developmental characteristics from both static and dynamic perspectives.

As shown in Table 5, the central region demonstrated the most notable static efficiency, with technical efficiency (TE = 0.9766), pure technical efficiency (PTE = 0.9877), and scale efficiency (SE = 0.9887) all approaching optimal levels. This can be attributed to balanced investment in provincial universities and management optimization under the “Rise of Central China” strategy. In contrast, the western region, despite achieving relatively high PTE (0.9794) in 2021, suffers from low SE (0.9261), resulting in significantly lower overall TE (0.9063). This reflects structural inefficiencies, with small, scattered, and weak institutions offsetting managerial advantages and keeping TE stagnant around 0.9.

**Table 5:** 2011–2021 disparities in regional higher education resource allocation efficiency levels.

Region	Efficiency Type	2011	2012	2013	2014	2015	2016	2017	2018	2019	2020	2021
Western	CTE	0.9588	0.9530	0.9415	0.9087	0.8985	0.9009	0.9177	0.9213	0.8877	0.8952	0.9063
	PTE	0.9693	0.9696	0.9607	0.9538	0.9447	0.9579	0.9711	0.9763	0.9491	0.9625	0.9794
	SE	0.9891	0.9832	0.9803	0.9527	0.9520	0.9414	0.9463	0.9445	0.9369	0.9306	0.9261
Central	CTE	0.9772	0.9795	0.9793	0.9681	0.9671	0.9863	0.9900	0.9932	0.9830	0.9682	0.9766
	PTE	0.9843	0.9857	0.9828	0.9784	0.9745	0.9896	0.9914	0.9949	0.9879	0.9795	0.9877
	SE	0.9927	0.9937	0.9962	0.9896	0.9925	0.9966	0.9986	0.9983	0.9948	0.9884	0.9887
Eastern	CTE	0.9198	0.9167	0.9149	0.9138	0.8950	0.9418	0.9331	0.9066	0.8956	0.9003	0.8961
	PTE	0.9624	0.9541	0.9626	0.9627	0.9423	0.9701	0.9779	0.9527	0.9464	0.9586	0.9424
	SE	0.9564	0.9614	0.9510	0.9489	0.9494	0.9710	0.9543	0.9523	0.9473	0.9401	0.9504
Northeastern	CTE	0.9040	0.9037	0.9030	0.8894	0.9158	0.9478	0.9741	0.9187	0.9310	0.9925	0.9923
	PTE	0.9084	0.9085	0.9086	0.8953	0.9338	0.9792	0.9872	0.9549	0.9785	1.0000	1.0000
	SE	0.9952	0.9948	0.9938	0.9933	0.9811	0.9676	0.9863	0.9621	0.9515	0.9925	0.9923

The eastern region exhibits diminishing returns to scale, with TE (0.8961) ranking lowest among all regions. Between 2015 and 2019, both TE and SE declined, likely due to resource dilution from excessive institutional expansion. Meanwhile, the northeastern region follows a unique efficiency trajectory: PTE reaches the theoretical optimum (1.0) after 2020, yet scale adjustment lags, highlighting structural contradictions during transition. Notably, the western region’s gradual recovery post-2015 aligns with the delayed effects of the “Western Development” strategy, while fluctuations in the eastern region’s PTE in 2017 may reflect initial resource misallocation under the “Double First-Class” initiative.

These findings offer critical policy implications; Eastern region should curb institutional overexpansion and enhance resource-sharing through regional university alliances. Western regions must address diseconomies of scale by adopting the central region’s balanced development model. The Northeastern region requires accelerated structural adjustment and modernization of traditional disciplines. Of particular concern is the efficiency decline in the eastern region despite high resource investment, signaling potential “involution” risks in higher education development. Proactive measures are needed to prevent inefficiency traps.

**Fig 4:**
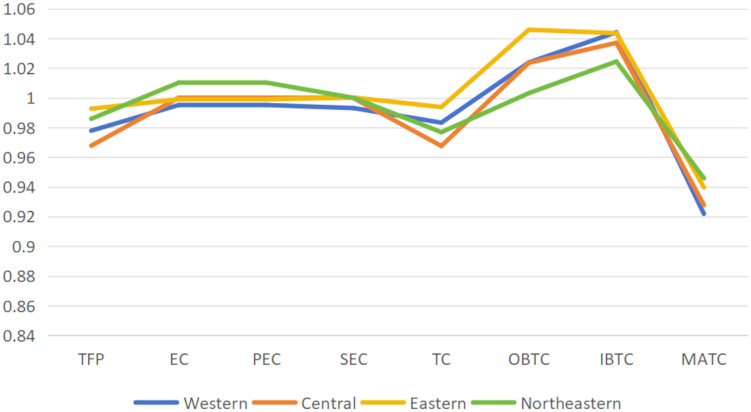
Mean changes in regional higher education resource allocation efficiency.

Combined with the Malmquist index and other methods, the study provides an in-depth analysis of the dynamic drivers behind these efficiency changes. Through the dynamic analysis of the allocation efficiency of higher education resources in the four major regions of China in Table 6 and Fig 4, we can clearly see the development characteristics and potential challenges in different regions.

**Table 6:** Mean changes in regional higher education resource allocation efficiency.

Region	TFP	EC	PEC	SEC	TC	OBTC	IBTC	MATC
Western	0.9779	0.9952	0.9952	0.9931	0.9833	1.0239	1.0442	0.9219
Central	0.9678	1.0001	1.0001	0.9997	0.9676	1.0236	1.0371	0.9278
Eastern	0.9927	0.9990	0.9990	1.0002	0.9938	1.0459	1.0436	0.9397
Northeastern	0.9859	1.0103	1.0103	1.0000	0.9769	1.0032	1.0245	0.9459

From the perspective of total factor productivity (TFP), from 20112021, the eastern region performed best overall (TFP = 0.9927) but still showed a slight recession. The challenges in the western region are most pronounced (TFP = 0.9779), and its technical efficiency (EC) and technological progress (TC) showing declines, forming a vicious circle of “technology-efficiency” double decline. This situation is directly linked to the insufficient R&D investment of western universities. Its technological change magnitude (match = 0.9219) is the lowest among the four regions, reflecting serious technological stagnation. It is worth noting that the west excels in the technological change of Investment Migration (IBTC = 1.0442). However, this investment-driven development model has not been effectively transformed into meaningful technological progress, highlighting the structural problems of resource allocation.

The central region shows the typical characteristics of “management optimization but insufficient innovation”. Its top performance in technical efficiency change (EC = 1.0001) demonstrates substantial improvements in management systems and resource allocation, such as optimizing the student–teacher ratio in Hubei Province to the best level nationally. However, its technological progress index (TC = 0.9676) is the lowest among the four regions, exposing shortcomings in basic research investment and the construction of major scientific research platforms in Central China. The situation in Northeast China is more complex. Its technological efficiency (EC = 1.0103) improved the fastest, but this increase may be mainly due to the statistical improvement brought about by structural reforms such as university mergers and professional mergers, rather than substantial management innovation, as evidenced by the continued decline of its technological progress index (TC = 0.9769).

From the perspective of the path choice of technological change, each region shows an clear trend of differentiation. The eastern region has significant advantages in technological change of output offset (OBTC = 1.0459), reflecting that colleges and universities in the region are better at transforming technological innovation into actual output. This advantage is closely related to the developed industrial base and achievement transformation mechanism in the Yangtze River Delta and Pearl River Delta. However, the eastern region’s technological progress index (TC = 0.9938) remains below 1, and its scale efficiency (SEC = 1.0002) is near a critical threshold, indicating that continued reliance on scale expansion could lead to “diseconomies of scale”. This serves as a warning for higher education development in the eastern region and suggests that the traditional model of scale expansion should be replaced with lightweight strategies such as “virtual teaching and research rooms”.

Given these regional characteristics, differentiated policy guidance is essential. The western region urgently needs to establish a “technology transfer alliance between the East and the west” to enhance the ability of technological innovation through joint laboratories and dual employment system of talents; The central region should set up a “special zone for basic research in Colleges and universities” to address shortcomings in original innovation. The eastern region needs to control the expansion of scale, shift towards connotative development, and incorporate technological progress indicators into the assessment system. Meanwhile, the northeast requires a more nuanced evaluation of its reforms, distinguishing between statistical improvements and substantive progress.

#### 4.3.2. Global regional comparative analysis.

Comparing and analyzing the efficiency trends of higher education in China’s regions relative to global development trend can provide a clearer positioning of China’s various regions in the global coordinate system and offer international reference for policymaking. The following comparison is made from three dimensions: efficiency level, evolution path, and reform practice.

***International coordinates of efficiency level and regional differentiation*:** The average TFP of higher education in China (0.9810) is basically on par with the average of emerging economies (0.983), but significantly lower than the average annual growth rate of 1.3% observed in OECD countries. This gap mainly comes from the lag in technological progress (TC): the TC in western China (0.9833) is only equivalent to 96.6% of similar universities in the United States (TC = 1.018), especially in terms of basic research conversion. The commercialization rate of patents in Chinese universities is 6.8%, far lower than the level of Stanford University in the United States (34%). The efficiency gap between the eastern and western regions of China (TFP difference of 1.48 percentage points) is similar to the gap between the core and peripheral countries of the European Union (Germany 1.025 vs Romania 0.981), but the speed at which the regional gap in China widens (an average of 0.12 percentage points per year) is faster than that in the European Union (0.05 percentage points), reflecting the need to improve the mechanism for regional coordinated development.

***Cross border comparison of evolutionary paths*:** The efficiency trap during the expansion phase, the “diseconomies of scale” phenomenon experienced in eastern China (with an average annual decline of 0.06% in SE), is highly similar to Brazil’s higher education expansion period from 2000 to 2010 (with a decline of 0.09% in SE). However, unlike the subsequent quality decline in Brazil, China has maintained its quality bottom line through the construction of “Double First Class”. This experience of balancing “scale quality” offers lessons forother BRICS countries. The technological degradation of MATC in western China (0.9219) is similar to that of Latin American countries such as Mexico (0.918), but better than that of South Africa (0.891). Unlike Latin American countries that rely on foreign investment for technology transfer, China’s western region has created a new paradigm for internal technology transfer among developing major countries through domestic technology diffusion (OBTC = 1.0239) supported by “counterpart support” program.

***International reference for reform practice*:** The German Excellence University Program has raised TE by 0.8 percentage points via a “concentration cluster” development model, offering guidance for breaking innovation bottlenecks in central China. Especially its “cross campus research center” mechanism can effectively solve the problem of fragmented scientific research in Chinese provincial universities.

The California public university system in the United States has evolved through tiered classification (research universities state colleges community colleges), maintaining a stable SE above 0.99 during the expansion period. The practice of functionally differentiated campuses offers direct reference for optimizing the spatial layout of universities in eastern China.

The operational efficiency of Israeli university technology transfer companies (such as Yissum) is 5.2 times that of China, and their “professor-broker-enterprise” tripartite cooperation mechanism is particularly suitable for improving OBTC in eastern China.

## 5. Analysis of factors influencing the efficiency of higher education resource allocation in the western China

### 5.1. Model selection and variable selection

While the DEA model has been used to measure the efficiency of resource allocation across western provinces, it is also necessary to explore which input factors influence these efficiency outcomes. To address this, the study employs the Tobit regression model, using the DEA-calculated efficiency scores as the dependent variable, to analyze the key factors that impact the efficiency of resource distribution.

The Tobit model is suitable for cases where the dependent variable is censored or truncated and related to the independent variables. Also known as a sample selection model or limited dependent variable model, it falls under the category of regression models for constrained dependent variables. Originally proposed by Nobel laureate James Tobin, this model has been further developed by many economists. Since the DEA-derived efficiency scores fall within the [0, 1] range and are truncated data, ordinary least squares estimation would be biased and inconsistent. Using the Tobit model, which is based on maximum likelihood estimation, minimizes biases from data limitations and high variance. Therefore, this paper utilizes the Tobit model for parameter estimation.

The allocation efficiency of higher education resources is a complex system influenced by multiple factors. As shown in the previous analysis, the allocation efficiency of higher education resources in the western region has not yet reached the DEA effective state. Therefore, it is important to explore the key influencing factors of its efficiency improvement.

When selecting independent variables, this study draws on the core concept of higher education resource allocation efficiency, relevant theory, and the practical realities of the western region. It considers the following dimensions to comprehensively reflect investment, environmental, and structural factors that affect efficiency, and to provide theoretical support.

**The proportion of education expenditure in government expenditure.** According to the resource dependence theory, public financial investment is the main source of higher education resources. In the western region, fiscal spending is essential for university funding. The national medium- and long-term education reform plan (2010–2020) explicitly sets the goal of education spending at 4% of GDP, underscoring the central role of financial investment. This proportion directly indicates government priority for education. Low proportions may mean insufficient resources or poor allocation structures that restrict efficiency; an appropriate proportion ensures basic investment and supports efficiency optimization. This variable thus serves as a key measure of core investment intensity.

**Per capita GDP.** New economic growth theory suggests that the level of economic development is the material basis for the development of higher education. Per capita GDP not only reflects the overall economic strength of the region, but also directly affects the income level and ability to pay residents. In the context of the relative backwardness of the western region, higher per capita GDP usually means that local governments have stronger financial capacity to support higher education; Families and individuals have stronger willingness and ability to share the cost of education and indirectly increase the total investment in higher education; Society can provide more resources and better job market, attract and maintain human and material resources. All these helps to improve the efficiency and efficiency of resource allocation.

**Regional gross domestic product (GDP).** According to macroeconomic theory, GDP measures the economic aggregate and market size of a region. A larger economy generates demand for high-quality talent and drives higher education development. A stronger industrial base offers more opportunities for university–industry collaboration and supports resource utilization efficiency. A stronger tax base will support larger public expenditure. Regional GDP and per capita GDP complement each other, describing the macroeconomic environment that affects allocation efficiency.

**Scale of higher education.** The scale of higher education directly influences its operation mode. At present, the western region has relatively few and small institutions compared to central and eastern China. The expansion of scale may bring economic of scale by spreading fixed costs across more students, supporting interdisciplinary integration, resource sharing, and optimizing internal resource allocation. Attract excellent teachers, scientific research teams and enterprise resources to form knowledge innovation clusters. Larger scale attracts top faculty and research teams, forming innovation clusters. This variable captures the structural characteristics of the system and its potential efficiency gains. In summary, this study selects the proportion of education expenditure in fiscal expenditure, per capita GDP, regional GDP, and scale of higher education as core independent variables. These four variables systematically cover the main aspects affecting allocation efficiency, from resource security and economic environment to structural system features, providing both theoretical and practical relevance. Together, they constitute a logical framework for analyzing the factors affecting efficiency.

### 5.2. Result analysis

This study uses a Tobit regression analysis to examine the factors influencing higher education resource allocation efficiency in the western provinces, using comprehensive technical efficiency, pure technical efficiency, and scale efficiency values derived from the DEA model as dependent variables. The detailed regression outcomes are presented in Table 7.

**Table 7:** Analysis of the influencing factors of the efficiency of higher education resource allocation in western China.

Variable	CTE	PTE	SE
The proportion of education expenditure in government expenditure	0.007***	0.124	0.077*
	(2.687)	(1.538)	(1.770)
Per capita GDP	0.046**	0.7	0.011**
	(1.996)	(0.385)	(2.549)
Regional GDP	0.398	0.024**	0.010**
	(0.845)	(2.250)	(−2.571)
Scale of higher education	0.324	0.029**	0.034**
	(−0.987)	(−2.184)	(2.125)
constant	0.000***	0.000***	0.000***
	(6.784)	(6.171)	(16.274)

Note: ***, **, and * represent the significant levels of 1%, 5% and 10%, respectively.

#### 5.2.1. The proportion of education expenditure in government expenditure.

The significant positive correlation between the proportion of educational expenditure in fiscal expenditure and comprehensive technical efficiency indicates that with the increase in the proportion of educational expenditure, higher education in the western region has received more attention and support from the government. This not only promotes the optimal allocation of educational resources but also enhances educational quality and activates research development. The increase in investment proportion has led to significant improvements in both comprehensive technical efficiency and scale technical efficiency. More financial input enables universities to introduce advanced teaching equipment, improve research facilities, attract and retain high-level faculty and researchers, thereby enhancing teaching and research capabilities and further improving the quality of educational output and academic achievements. For example, Guizhou Province has continuously increased fiscal investment in higher education in recent years, with a significant growth in per-student funding, effectively supporting multiple universities such as Guizhou University and Guizhou Normal University in expanding campuses, purchasing advanced experimental equipment, expanding disciplines, and establishing new colleges. Such actions have expanded enrollment scales and enhanced overall teaching and research capabilities, demonstrating the direct promotion of “quantitative” resource increases on scale expansion and basic capacity building.

However, regarding pure technical efficiency, even with the government’s increased emphasis on higher education, its efficiency improvement is not significant. This may be because pure technical efficiency more profoundly reflects management and operational efficiency, involving the issue of maximizing output under existing resource conditions. This does not solely rely on financial investment but also requires effective management strategies, innovative teaching methods, and scientific educational policies. Although fiscal expenditure has increased, if the educational management system is rigid, educational policies are not implemented effectively, or educational resources are unevenly distributed, it may not effectively translate into specific improvements in educational implementation efficiency. For instance, despite increased investment in some western provinces, universities still face problems such as multiple administrative levels, cumbersome approval processes, clear barriers to educational resources among different departments within the university, and low sharing rates. This reflects structural and management issues in resource allocation, where investment has not been effectively transformed into management optimization and fine-grained resource utilization.

Therefore, for policymakers and managers in higher education, while increasing educational investment, it is necessary to further optimize educational policies, reform the management system, improve resource allocation efficiency, and strengthen educational quality monitoring to ensure that investment is translated into practical improvements in educational quality and efficiency. While ensuring total investment, deepen the reform of the management system, promoting the “delegation of powers, improvement of regulation, and optimization of services” reform in universities, simplifies administrative processes, and grant universities greater autonomy. Strengthen the performance orientation of investment, link part of the fiscal allocation with PTE (Pure Technical Efficiency) indicators such as the optimization degree of student-faculty ratio, equipment utilization rate, scientific research achievement transformation rate, and curriculum resource sharing rate, to guide universities to pay attention to resource use efficiency. Promote internal integration and sharing of resources, encourage universities to establish university-level large-scale instrument sharing platforms and online curriculum resource libraries, break down departmental barriers, and improve resource utilization efficiency.

#### 5.2.2. Per capita GDP.

The significant positive correlation between per capita GDP and comprehensive technical efficiency reveals the positive impact of economic growth on education and technological development. As per capita GDP increases, household investment in education correspondingly rises, especially in western regions, leading to more social funds flowing into the field of higher education. This growth not only improves educational infrastructure but also enhances academic resources and research funding, thereby contributing to the improvement of teaching quality and research capabilities in educational institutions. These changes directly promote the enhancement of comprehensive technical efficiency and scale technical efficiency, manifested in more students and research projects meeting higher academic and technical standards. For example, universities in Shaanxi Province, with relatively higher per capita GDP, attract significantly more social donations and university-enterprise cooperation projects than those in Qinghai Province. Universities like Xi’an Jiaotong University have utilized social funds to establish multiple joint laboratories and internship bases, supplementing government investment, improving facilities, expanding service capabilities, and enhancing overall strength.

However, although the increase in social funds has brought quantitative expansion and some quality improvement to higher education, this growth has not significantly promoted technological progress, and its role in enhancing pure technical efficiency remains limited. Pure technical efficiency more profoundly relies on the efficiency and innovation of educational management, involving how to maximize output within the existing resource framework. This requires higher education institutions not only to increase investment but, more importantly, to optimize management structures, enhance teaching methods and research quality, and achieve effective allocation of internal resources. To truly improve pure technical efficiency, policymakers and managers in higher education need to focus on formulating more effective educational strategies and management models, such as promoting lean teaching, strengthening the integration of academic research and practice, and using high-tech tools and methods to optimize the educational process. Additionally, encouraging and supporting educational innovation projects, such as interdisciplinary research and international cooperation, is also a key strategy for enhancing the pure technical efficiency of higher education systems. These measures will help improve the overall efficiency and quality of the education system, ultimately achieving a comprehensive improvement in technical efficiency.

Therefore, for policymakers and managers in higher education, universities should actively expand diversified financing channels, improve donation tax incentives, and encourage alumni, enterprises, and social forces to donate to education. It is also necessary to deepen the integration of industry and education as well as university-enterprise cooperation, attracting enterprises to invest in building joint R&D centers and training bases. Meanwhile, social capital should be guided to invest in key areas for efficiency improvement, such as supporting teaching reform and innovation projects (e.g., blended teaching reform and interdisciplinary curriculum development), carrying out smart campus management system construction, implementing management efficiency improvement projects, and building shared platforms that can significantly enhance resource utilization rates.

#### 5.2.3. Regional gross domestic product (GDP).

The significant positive correlation between regional GDP and pure technical efficiency indicates that as regional economies grow, higher education institutions gain more resources to advance educational technology and management innovation. These additional resources are typically utilized to enhance education quality and efficiency through the introduction of advanced teaching technologies, updating course content, and strengthening faculty training. Such improvements directly boost the technical efficiency of educational institutions, which refers to maximizing output under given resource conditions. For instance, Sichuan Province, which leads in GDP among western regions, supports local universities in significantly investing in smart education development, promoting online teaching platforms and virtual simulation experiments, thereby optimizing teaching processes and management, and substantially enhancing output capacity under given resource conditions. Additionally, the Chongqing Municipal Government established a special fund to support higher education management innovation research and pilot projects, aiming to improve pure technical efficiency. However, despite the increase in funds and resources brought about by rising regional GDP, this does not necessarily lead to an improvement in scale technical efficiency. Scale technical efficiency measures whether higher education institutions can maintain or enhance efficiency during expansion. Research shows that relying solely on scaling strategies does not always effectively enhance efficiency. In fact, excessive expansion can sometimes result in management inefficiency and resource wastage. For example, during a period of rapid GDP growth, Yunnan Province blindly approved the upgrading or expansion of several local colleges, leading to shortages in faculty, a sharp increase in student-to-faculty ratios, inadequate management, and low utilization rates of new campuses, thereby reducing scale efficiency. Therefore, under the impetus of GDP growth, higher education institutions increasingly focus on improving internal quality and management rather than simply pursuing scale expansion, which negatively impacts scale technical efficiency.

Furthermore, regional GDP growth has little effect on overall technical efficiency, possibly because overall technical efficiency is influenced not only by funding and resource levels but also by policy, management, regional economic structure, and other external environmental factors. Thus, even though regional economic growth provides more investments for educational institutions, enhancing overall technical efficiency requires broader strategies and reform measures. In summary, regional GDP growth provides financial support for technological and managerial innovation in higher education, thereby promoting improvements in pure technical efficiency. However, this growth negatively impacts scale efficiency because higher education institutions begin to prioritize enhancing internal efficiency and quality over reckless expansion. This shift underscores the importance of high-quality development, and understanding these dynamics and their underlying factors is crucial for education policymakers.

Therefore, for higher education policymakers and administrators, it is crucial to leverage the dividends of economic growth by establishing special funds aimed at improving quality and efficiency. Provincial finances should allocate dedicated funds to provide targeted support for universities in areas such as educational and teaching reforms, process reengineering, informatization construction, and the development of resource-sharing platforms, thereby directly enhancing pure technical efficiency. Growth in scale should be cautiously controlled, with a stronger emphasis on quality and efficiency evaluations. A rigorous review and benefit assessment mechanism should be established for university expansion, prioritizing the support for connotative development. Existing universities are encouraged to adapt to changing demands by optimizing internal structures and utilizing existing resources effectively, rather than simply expanding. Additionally, fostering regional university alliances and resource sharing should be promoted, breaking down administrative boundaries to establish inter-provincial or intra-provincial university networks. These alliances should collaborate deeply in areas such as course selection, credit recognition, faculty exchanges, and shared use of large-scale equipment, thereby improving overall resource utilization efficiency and enhancing regional comprehensive technical efficiency.

#### 5.2.4. Scale of higher education.

The expansion of higher education scale has different impacts on various types of technical efficiency. First, there is a positive correlation between scale efficiency and the size of education, indicating that as the scale of educational institutions increases, the economies of scale in production and operation are realized. This typically means that large-scale operations can reduce unit costs, improve resource utilization efficiency, and thus promote the overall improvement of educational efficiency. Large educational institutions can accommodate more students, utilize larger facilities and equipment, and these usually bring cost benefits as the scale increases. Some comprehensive universities, such as Lanzhou University and Sichuan University, with their large student bases, have relatively low per capita costs in libraries, sports facilities, and logistics services, and the utilization rates of large-scale instruments and equipment are more likely to reach saturation, demonstrating the advantages of educational scale.

However, from the perspective of pure technical efficiency, the expansion of higher education scale shows a negative impact. Pure technical efficiency primarily focuses on the optimal allocation of resources and management efficiency. When the scale of educational institutions expands rapidly, it may lead to increased complexity in management, higher coordination and supervision costs, thereby affecting educational quality and teaching efficiency. This effect is manifested as a relative lag in educational management and teaching levels, a deterioration in the teacher-student ratio, delayed management decisions, and unequal distribution of educational resources. For example, some local universities in western regions, after expanding their enrollments, experienced a sharp increase in management scope, resulting in distorted information transmission, delayed decision-making, inadequate teaching supervision, difficulties in interdepartmental coordination, and internal unfairness in resource distribution, severely damaging pure technical efficiency.

In addition, regarding overall technical efficiency, the expansion of higher education scale does not seem to have a significant impact. Overall technical efficiency considers the combined performance of scale efficiency and pure technical efficiency. While the expansion of scale may enhance scale efficiency, it may simultaneously reduce pure technical efficiency, and the effects of these two may offset each other, leading to no significant change in overall efficiency.

Therefore, for policymakers and managers in higher education, the key lies in finding an appropriate balance, that is, achieving a reasonable match between scale expansion and improvements in management and teaching quality, ensuring that educational expansion can proceed without sacrificing teaching and management efficiency. This may require adopting innovative management strategies, improving management efficiency, and optimizing resource allocation to achieve dual enhancements in educational quality and scale. Establishing principles of moderate scale and optimized structure, guiding universities to determine their optimal scale based on their positioning, faculty strength, management capabilities, and local needs, avoiding blind pursuit of size. Strengthening modern governance within universities, promoting the improvement of internal governance structures, implementing flat management, and utilizing information technology to enhance the precision of management and decision-making efficiency to address the complexities brought by scale. Establishing a dynamic resource allocation mechanism based on colleges/disciplines, granting departments greater authority in resource allocation, while establishing a competitive internal resource allocation and dynamic adjustment mechanism based on performance and needs, improving the accuracy and responsiveness of resource distribution, and alleviating the negative impact of scale expansion on pure technical efficiency.

## 6. Conclusion, recommendation and limitations

### 6.1. Conclusion

This study assessed the efficiency of higher education resource allocation in western China from 2010 to 2020 using DEA-Malmquist and Tobit models for both static and dynamic analyses. The static results indicate that although the average TE, PTE, and SE values are relatively high, they still fall short of the efficiency frontier. The dynamic analysis further shows that the annual average TFP remains below 1, indicating an overall decline in efficiency and rendering DEA ineffective. Additionally, TFP values in various regions of western China are also below 1, reflecting widespread inefficiency and significant regional disparities.

Empirical analysis using the Tobit model revealed significant positive relationships between the proportion of education spending in fiscal expenditure, per capita GDP, and comprehensive technical efficiency. These factors also positively influenced scale technical efficiency. On the other hand, regional GDP showed a strong positive correlation with pure technical efficiency but negatively impacted scale efficiency. Additionally, the scale of higher education was positively associated with scale efficiency while negatively affecting pure technical efficiency.

### 6.2. Recommendation

First, optimize the structure of education expenditure by increasing investment in areas with low scale efficiency to improve technical efficiency and generate positive effects through compounding benefits. Colleges and universities should prioritize allocating more funds to educational expenses while maintaining the necessary internal conditions and scale to accelerate improvements in educational efficiency. Second, financial support for higher education in western China should be increased by raising the proportion of education expenditure within the fiscal budget. More funds should be directed to education, raising per capita funding standards for universities in western China, and developing region-specific funding policies based on the actual needs of universities. Special financial support should be given to areas such as Ningxia, Qinghai, and Tibet to boost local higher education efficiency, while regions with higher efficiency should be incentivized through rewards to narrow the funding gap.

Third, alongside emphasizing the input of talent, financial, and material resources, more focus should be placed on enhancing the “quality” of education. Given the significant changes in China’s demographic trends, attention should be paid not only to improving scale efficiency but also to aligning the development goals of universities with local needs. This requires elevating the management and technological capabilities of universities, shifting the emphasis from expanding “quantity” to enhancing “quality.” The intrinsic development of higher education in western China demands not only an increase in “hard inputs” but also deeper cultivation in areas such as systems, governance, and mindset, promoting internal growth and capability enhancement. Fourth, focus on aligning educational quality with market demands. The development of higher education should prioritize matching educational offerings with market needs, avoiding indiscriminate expansion. Institutions should adjust their scale and curriculum flexibly to swiftly respond to shifts in market demands, not only expanding capacity but also improving internal quality. This will help cultivate talent that meets future workplace demands, ensuring the efficient allocation of resources and achieving an optimal match between education and market needs.

Fifth, considering the overall decline in the efficiency of higher education resource allocation in the western regions, a detailed and dynamic monitoring mechanism should be established to promote sustainable educational development. Regular assessments of resource allocation efficiency will provide accurate data on resource utilization, help identify issues early, and allow for more targeted and effective policy adjustments. Through systematic improvements and ongoing evaluations, higher education resource allocation efficiency in western China can be gradually enhanced, supporting the region’s economic and social progress.

### 6.3. Limitations and future research directions

Firstly, the research results may be limited the study’s geographic focus. Our study was conducted in the unique context of China, and it is necessary to consider extending the research to other developing countries.

Secondly, factors that affect the efficiency of higher education resource allocation may include many external environmental factors that are difficult to quantify, such as policy changes, which may be difficult to accurately measure and incorporate into the models. These areas require further research and validation.

Thirdly, our research data is limited to provincial panel data. If internal data from universities can be obtained, future research on the efficiency of higher education resource allocation can be more in-depth and intuitive. For example, using discipline-level data such as per capita funding, experimental equipment utilization rate, and research achievement conversion rate to calculate the technical efficiency of each discipline (DEA model) and identify which disciplines have resource redundancy or insufficiency.

Fourthly, although the DEA Malmquist index can measure efficiency, high efficiency does not necessarily mean high- education quality. Whether the effective allocation of educational resources truly translates into the improvement of educational quality requires further research and verification. For example, designing mixed research that combines efficiency analysis with quality assessment (such as classroom observation and student literacy assessment) to establish an “efficiency quality” correlation model; Track the long-term changes in educational outcomes of high-efficiency units and distinguish between short-term efficiency and long-term quality effects.

Fifth, one area that remains partially addressed is the discussion of robustness and sensitivity analysis. This can be further strengthened through the following strategies: Bootstrap DEA analysis to assess the stability of efficiency scores; alternative second-stage models(e.g., truncated regression, SFA) for comparative analysis with the Tobit model; sensitivity tests on indicator specification or weight constraints; and temporal sub-sample comparisons (e.g., pre- and post-2016 policy shifts).Future research should prioritize these methodological refinements to enhance the robustness and credibility of empirical outcomes.

## Supporting information

S1 File Data on higher education in western China. (RAR)
